# Evolutionarily Established Palmitoylation-Dependent Regulatory Mechanisms of the Vertebrate Glutamatergic Synapse and Diseases Caused by Their Disruption

**DOI:** 10.3389/fnmol.2021.796912

**Published:** 2021-11-15

**Authors:** Takashi Hayashi

**Affiliations:** Biomedical Research Institute, National Institute of Advanced Industrial Science and Technology (AIST), Tsukuba, Japan

**Keywords:** palmitoylation, glutamate receptor (GluR), PDZ protein, excitatory synapse, vertebrate, eutherian, evolution

## Abstract

Glutamate is the major excitatory neurotransmitter in the vertebrate brain and various modifications have been established in the glutamatergic synapses. Generally, many neuronal receptors and ion channels are regulated by *S*-palmitoylation, a reversible post-translational protein modification. Genome sequence databases show the evolutionary acquisition and conservation concerning vertebrate-specific palmitoylation of synaptic proteins including glutamate receptors. Moreover, palmitoylation of some glutamate receptor-binding proteins is subsequently acquired only in some mammalian lineages. Recent progress in genome studies has revealed that some palmitoylation-catalyzing enzymes are the causative genes of neuropsychiatric disorders. In this review, I will summarize the evolutionary development of palmitoylation-dependent regulation of glutamatergic synapses and their dysfunctions which are caused by the disruption of palmitoylation mechanism.

## Introduction

Vertebrates comprise a lot of animal species classified within the subphylum Vertebrata, which consists of a majority of the phylum Chordata. Variety of species in mammals, birds, reptiles, amphibians, lobe-finned fishes, ray-finned fishes, cartilaginous fishes, and jawless fishes belong to vertebrates. They have diverged from the common ancestral chordate. Other subphyla Cephalochordata and Urochordata in the phylum Chordata are composed of lancelets and sea squirts (also known as ascidians), respectively. The vertebrate brain has been naturally developed from the primitive chordate nervous system. Almost as if a language is deeply rooted in the accumulated history of speakers (Cavalli-Sforza, 2000), essential structure, operating principles, and components of vertebrate brain basically hold the evolutionary accumulation in the history of life. Regarding many vertebrate synaptic protein orthologs including ion channels and neurotransmitter receptors, these component parts commonly exist in the whole chordates as well as species in other animal phyla. In this review, I will discuss recent findings supporting the evolutionarily developed modification in the vertebrate brain and will address their functional significance.

## Evolutionary Acquisition and Conservation of Synaptic Palmitoylation in The Vertebrate Lineage

Among various neurotransmitters available in animals, glutamate was evolutionarily selected as the major excitatory neurotransmitter in the vertebrate central nervous system. Thus, glutamate receptor (GluR) family proteins play central roles in excitatory synaptic transmission and plasticity in the vertebrate brain (Shepherd and Huganir, 2007; Kessels and Malinow, 2009; Anggono and Huganir, 2012; Diering and Huganir, 2018). GluRs are composed of ionotropic and metabotropic GluRs. The ionotropic glutamate receptors (iGluRs) are pharmacologically and electrophysiologically classified into four groups, α-amino-3-hydroxy-5-methyl-4-isoxazolepropionate (AMPA)-type, kainate (KA)-type, δ-type, and *N*-methyl-D-aspartate (NMDA)-type receptors, basically named after their selective agonist drugs (Seeburg, 1993; Hollmann and Heinemann, 1994; Mori and Mishina, 2003). Ancestral animal GluR genes are supposed to be developed from prokaryotic potassium-selective ion channel GluR0 (Arinaminpathy et al., 2003).

Essential biological functions of proteins are basically determined by the amino acid sequence of proteins. Moreover, post-translational protein modifications (PTPMs) enable precise and dynamic control of protein localization, membrane trafficking, and fine-tuning of protein functions. In many types of PTPMs, protein *S*-palmitoylation is a prominent type of fatty acylation, characterized by the reversible covalent attachment of lipid palmitate on target proteins (Resh, 2016). Palmitate is a saturated fatty acid, which most abundantly exists in the vertebrate brain as well as in the whole body. The biochemical process of *S*-palmitoylation is enzymatically mediated transfer of palmitoyl group from palmitoyl-coenzyme A (palmitoyl-CoA) to intracellular cysteine residues of target proteins *via* thioester bonds. Accumulating genome information in animal species increasingly clarifies that low rates of substitution occur at structurally or functionally significant amino acid residues during molecular evolution against continuous mutation pressure.

**Figure 1:**
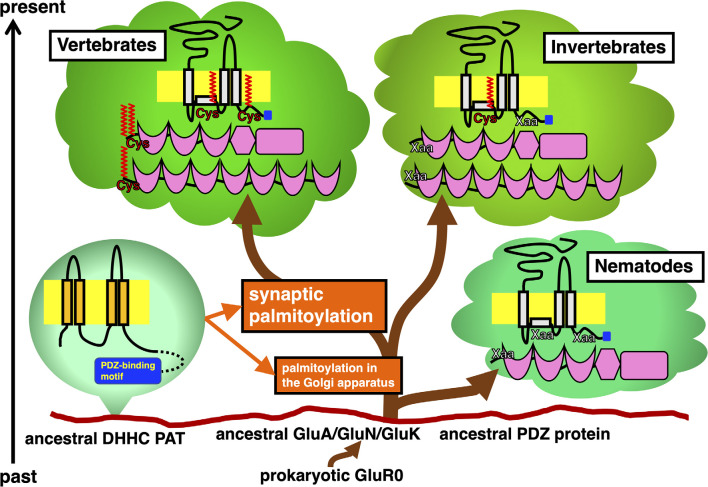
Timeline of appearance of GluR and PDZ protein orthologs in the animal phylogenetic tree diverged from the same primitive root on the eukaryotic ground. Stepwise establishment of GluR palmitoylation in the Golgi apparatus and synaptic palmitoylation in vertebrate GluRs and PDZ proteins are shown in orange. DHHC PATs, which evolutionarily expand, catalyze these modifications. Ancestral AMPA receptor GluA, NMDA receptor GluN, and KA receptor GluK had been developed from the prokaryotic GluR0. Divergence of orthologs of these GluRs and PDZ proteins in vertebrate and invertebrate lineages are shown. Schematic of these proteins indicate the location of palmitoylation sites (colored in red) and PDZ-binding motif (colored in blue).

**Figure 2:**
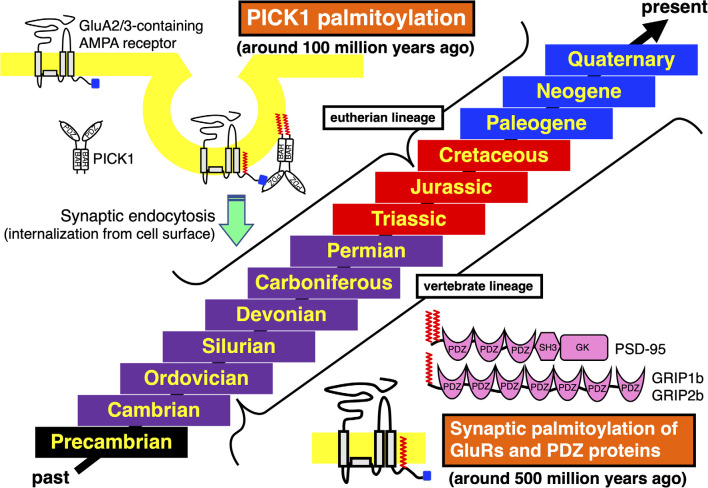
Acquisition and conservation of vertebrate-specific palmitoylation in GluRs and PDZ proteins and eutherian-specific palmitoylation in PICK1. Geologic time scale from the past to the present is shown. The Phanerozoic Eon, during which abundant animal species have expanded and became partly extinct, is majorly classed into the Paleozoic Era (Cambrian, Ordovician, Silurian, Devonian, Carboniferous, and Permian periods, marked in purple), the Mesozoic Era (Triassic, Jurassic, and Cretaceous periods, marked in red) and the Cenozoic Era (Paleogene, Neogene, and Quaternary periods, marked in blue). The preceding Precambrian period is marked in black. Synaptic palmitoylation was established only in the common vertebrate ancestors of GluRs, PSD-95, GRIP1b, or GRIP2b around 500 million years ago in the late Cambrian to the early Ordovician periods, which has been almost completely conserved in the whole vertebrate lineage. Moreover, PICK1 palmitoylation has been conserved in most eutherian lineage, which was acquired in the common ancestor of eutherian species belonging to the superorders Xenarthra, Laurasiatheria, Euarchontoglires, and the orders Sirenia and Proboscidea around 100 million years ago in the early Cretaceous period.

Previous studies have shown the vertebrate-specific conservation of palmitoylated cysteine residues in GluRs orthologs (Figure 1). Concretely, synaptic palmitoylation sites in AMPA receptor subunits GluA1, GluA2, GluA3, and GluA4 (also known as GluR1-4, GluRA-D, or GluRα1-4) orthologs (Hayashi et al., 2005; Itoh et al., 2018, 2019; Iizumi et al., 2021), NMDA receptor regulatory subunits GluN2A and GluN2B (also known as NR2A and NR2B or GluRε1 and GluRε2) orthologs (Hayashi et al., 2009; Mattison et al., 2012) and KA receptor subunit GluK2 (also known as GluR6 or GluRβ2) orthologs (Pickering et al., 1995) are almost completely conserved only in the vertebrate lineage (Hayashi, 2014, 2021). AMPA receptors and NMDA receptors are palmitoylated at their two distinct sites (Hayashi et al., 2005, 2009; Hayashi, 2021). Another palmitoylation site causes the receptors to accumulate in the Golgi apparatus (Figure 1). Cysteine residues at the corresponding sites are widely conserved in both vertebrates and invertebrates besides nematodes (Thomas and Hayashi, 2013). This Golgi apparatus localizing palmitoylation thus appears to act as a “quality control” mechanism to ensure correct receptor maturation. In contrast, vertebrate-specific synaptic palmitoylation sites in AMPA receptors and NMDA receptors are implicated in “quantitative regulation” of synaptic strength, which relate to complex neuronal events such as long-lasting synaptic plasticity. Evolutionarily, the sudden appearance of many animal phyla had occurred in the Cambrian period. These data strongly indicate that synaptic palmitoylation-dependent GluRs regulation had established just after the divergence of the common vertebrate ancestor from chordates around 500 million years ago in the late Cambrian to the early Ordovician periods (Figure 2). The GluR palmitoylation mechanisms have been evolutionarily conserved against mutation pressure throughout whole vertebrate species, presumably because the protein palmitoylation plays essential and irreplaceable roles for vertebrate-specific excitatory synaptic functions. Extremely conserved synaptic palmitoylation sites in the vertebrate lineage from jawless fishes to humans indicate that this modification may form the basis of vertebrate-specific brain functions.

Similarly, vertebrate-specific conservations of palmitoylation sites are also observed in other ion channels and neurotransmitter receptors (Borroni et al., 2016), such as HCN2 (hyperpolarization-activated cyclic nucleotide-gated channel 2) orthologs (Itoh et al., 2017), a water channel AQP4 (aquaporin family protein 4) orthologs (Hayashi, 2017), and serotonin (chemically known as 5-hydroxytryptamine, 5-HT) receptors 5-HT_1A_, 5-HT_4_, 5-HT_7_ receptor orthologs (Kaizuka and Hayashi, 2018).

## Stepwise Refinement of Palmitoylation-Dependent Regulation of GluR-Binding Proteins

As the major excitatory neurotransmitter receptors, GluRs ensure the minimal and essential excitatory synaptic transmission in the vertebrate brain. Furthermore, additional participation of GluR-binding proteins greatly refines temporal and spatial control of excitatory synaptic functions (Thomas and Hayashi, 2013). The most well-studied palmitoylated GluRs regulators are PDZ (PSD95/Dlg1/zo-1) domain-containing proteins, such as PSD-95 (postsynaptic density protein 95), GRIP1 (GluR-interacting protein 1), GRIP2, and PICK1 (protein interacting with C-kinase 1), which make complex with GluRs, respectively. These vertebrate PDZ domain proteins have orthologs in other phyla (Figure 1).

PSD-95 binds to NMDA receptor subunits GluN2A-2D and KA receptor subunit GluK2 through its PDZ domain. PSD-95 also interacts with AMPA receptors-transmembrane AMPAR regulatory proteins (TARPs) complex and regulates AMPA receptors ion channel properties as well as their membrane localization in many animal species. Orthologs of PSD-95, also known as SAP-90 (synapse-associated protein 90), broadly exist in vertebrates and many invertebrates. The fruit fly *Drosophila melanogaster* protein Discs large (DLG) and a nematode *Caenorhabditis elegans* protein DLG-1 are orthologs of vertebrate PSD-95. While vertebrate PSD-95 possess N-terminal palmitoylated cysteine residues, invertebrate PSD-95 orthologs lack these motifs at the corresponding site. N-terminal palmitoylation sites are lost in DLG orthologs from primitive chordates (e.g., an ascidian *Ciona intestinalis*, sometimes known by the common name of vase tunicate) and hemichordates (e.g., an acorn worm *Saccoglossus kowalevskii*). Therefore, synaptic regulation through PSD-95 palmitoylation appears unique to vertebrates (Figure 1).

Other PDZ proteins GRIP1a and its related GRIP2a/ABP-L (AMPA receptor-binding protein-L), are alternatively spliced to generate palmitoylated isoforms, GRIP1b, GRIP2b/pABP-L. PDZ domains of these proteins interact with GluA2 C-terminus. The neuronal trafficking and localization of vertebrate AMPA receptor complexes can be regulated by these binding partners *via* palmitoylation (Thomas et al., 2012). The GRIP1b N-terminal palmitoylation sequence is widely present in vertebrate genomes. However, GRIP1 sequences in primitive chordates (*C. intestinalis*) and hemichordates (*S. kowalevskii*) lack this palmitoylation site. Generally, palmitoylation motifs are lost in invertebrate GRIP1 and GRIP2 orthologs including insects. Although the dGRIP, a *Drosophila* GRIP1 ortholog, exceptionally contains a cysteine residue at the corresponding position to vertebrate GRIP1, amino acids around the dGRIP cysteine are poorly conserved compared with vertebrate GRIP1b. Thus, it remains unclear whether dGRIP can serve similar functions to mammalian GRIP1b even if dGRIP is palmitoylated. Palmitoylation of iGluRs, PSD-95, GRIP1b, GRIP2b, HCN2, AQP4, and 5-HT receptor orthologs seem to be acquired in the ancestor of vertebrates at about the same time around 500 million years ago (Figure 2).

The PDZ domain of PICK1 competitively binds to the AMPA receptor subunit GluA2 C-terminus (Figure 2). PICK1 controls GluA2-containing AMPA receptor endocytosis from the synaptic surface (Shepherd and Huganir, 2007; Thomas et al., 2013). Sequence databases show that the PICK1 C-terminal palmitoylation is limited in some orders in mammals (Hayashi, 2015). In contrast to the evolutionary establishment of palmitoylation in other neuronal protein orthologs, widely and specifically recognized in vertebrates as mentioned above, palmitoylation of PICK1 had been acquired step by step along the divergence of mammalian species. The class Mammalia consists of three living groups, the subclasses Prototheria (e.g., platypus), Metatheria (extant marsupials, such as gray short-tailed opossum and wallaby), and Eutheria (extant placentals). PICK1 orthologs in both subclasses Prototheria and Metatheria lack palmitoylation sites. Placental mammals are further classified into the four major superorders, Afrotheria, Xenarthra, Laurasiatheria, and Euarchontoglires (Springer and Murphy, 2007). All these eutherian (placental) species diverged from the same root around 100 million years ago in the early Cretaceous period (Springer et al., 2011). In the superorder Afrotheria, aardvark PICK1 ortholog shows the primitive C-terminal sequence (-Gly-Ser in the order Tubulidentata). Some afrotherian PICK1 orthologs have additional short Cys non-containing motif to the anticipated original (-Gly-Ser-Trp-Xaa-Gly-Ser in the order Macroscelidea, e.g., Cape elephant shrew and in the order Afrosoricida e.g., Cape golden mole, lesser hedgehog tenrec). The Cys-containing PICK1 palmitoylation motif is found in species belonging to the superorders Afrotheria (-Gly-Ser-Trp-Cys-Gly-Ser in the order Sirenia, e.g., Florida manatee and in the order Proboscidea, e.g., African elephant), Xenarthra (-Gly-Ser-Trp-Cys-Gly-Ser in the order Cingulata, e.g., armadillo), Laurasiatheria (-Gly-Ser-Trp-Cys-Asp-Ser, e.g., dog) and Euarchontoglires (-Gly-Ser-Trp-Cys-Asp-Ser, e.g., mouse, human). The PICK1 palmitoylation-dependent refined control of AMPA receptors should originate in the common ancestor of these eutherian (placental) species (Hayashi, 2015). This improved PICK1 regulation mechanism has been broadly conserved during evolution in the extant eutherian (placental) lineage, whereas a cysteine residue at the corresponding site is lost in some exceptional species such as hedgehog (the order Erinaceomorpha in the superorder Laurasiatheria: -Gly-Ser-Trp-Ser-Asp-Ser) and American pika (the order Lagomorpha in the superorder Euarchontoglires: -Gly-Ser-Trp-Ser-Asp-Ser). Furthermore, PICK1 orthologs in all reported species in the family Bovidae (cattle, yak, water buffalo, sheep, goat, antelope: -Gly-Ser-Trp-Cys-Asn-Ser) possess different Cys-containing sequences from the standard eutherian motif, which may imply ongoing molecular evolution of PICK1 palmitoylation (Hayashi, 2015).

## Enzymes Catalyzing Palmitoylation in Neurodegenerative Disease and Mental Disorders and Their Evolutionary Expansion

Biochemical researches have revealed that cycles of palmitoylation by palmitoyl acyl transferases (PATs) and depalmitoylation by palmitoyl-protein thioesterases (PPTs) serve as a cellular regulatory mechanism from yeast to human (Fukata and Fukata, 2010; Matt et al., 2019). Presently, 23 human and 24 mouse genes have been identified in a PAT family containing the Asp-His-His-Cys (DHHC) catalytic motif within a cysteine-rich, zinc finger-like domain (Korycka et al., 2012; Gottlieb and Linder, 2017; De and Sadhukhan, 2018). As PPTs, acyl-protein thioesterase (also called as Lypla) 1 and 2 (APT1 and APT2), APT-like, palmitoyl-protein thioesterase 1 and 2 (PPT1 and PPT2), and 17 α/β-hydrolase domain-containing family proteins are currently known (Lin and Conibear, 2015; Yokoi et al., 2016). About 40% of PATs are known to be linked to human diseases (Chavda et al., 2014). Palmitoylation deficiency leads to neuropsychiatric disorders. Especially, mutations in DHHC PATs are related to neurogenerative diseases (DHHC7, DHHC12, and DHHC21 in Alzheimer’s disease, DHHC17/HIP14 (huntingtin-interacting protein 14) and DHHC13/HIP14L (HIP14-like) in Huntington’s disease) and mental disorders (DHHC5 in bipolar disorder, DHHC8 in schizophrenia, DHHC9, and DHHC15 in X-linked intellectual disability).

Comparison of animal genomes suggests that DHHC PATs homologs seem to have expanded during animal evolution. PDZ-binding motifs are present in C-termini of several DHHC PATs, which are recognized by PDZ domain-containing proteins (Figure 1). Predicted C-terminal PDZ-binding motifs on DHHC PATs are increasingly recognized in DHHC PAT homologs (Thomas and Hayashi, 2013): a nematode worm (*C. elegans*, 6.7%), fruit fly (*D. melanogaster*, 18.2%), Zebrafish (*Danio rerio*, 36.4%), frog (*Xenopus tropicalis*, 38.1%), mouse (*Mus musculus*, 33.3%), and humans (*Homo sapiens*, 39.1%). The proportion of PDZ-binding PATs goes up from 6.7% in worms to around 36.7% in vertebrates as DHHC PAT numbers increase. All eight PDZ-binding mouse PATs (DHHC3, DHHC5, DHHC7, DHHC8, DHHC14, DHHC16, DHHC17, and DHHC21 orthologs) are detected in 14 neuronal tissue-expressing PATs (57.1%), indicating that PDZ-dependent interaction of PATs is likely to play crucial roles in the vertebrate brain.

## Conclusion and Perspective

So far, there is no direct evidence that implicates mutations at the palmitoylation sites of iGluRs and that their binding proteins cause any neuropsychiatric disorders. However, palmitoylation of these orthologs was established in the very early stage of vertebrate evolution and they have been extremely well conserved in the whole vertebrate species or in specific eutherian lineage as summarized in this review. This essential synaptic modification system may be protected by multiple layers of regulatory mechanisms to prevent disturbance of vertebrate brain functions. Further accumulation of genome sequence data will fill in the blanks of the animal order, family, and genus list concerning vertebrate divergence. Additional information will reveal the timeline of establishment and divergence of these palmitoylation sites-containing motifs in more detail. Especially, sequence information on urochordates (sea squirts or ascidians), cephalochordates (lancelets), and cyclostomes (lampreys and hagfishes) will clarify the initial acquisition of the mechanism of synaptic palmitoylation.

## Author Contributions

The author confirms being the sole contributor of this work and has approved it for publication.

## Conflict of Interest

The author declares that the research was conducted in the absence of any commercial or financial relationships that could be construed as a potential conflict of interest.

## Publisher’s Note

All claims expressed in this article are solely those of the authors and do not necessarily represent those of their affiliated organizations, or those of the publisher, the editors and the reviewers. Any product that may be evaluated in this article, or claim that may be made by its manufacturer, is not guaranteed or endorsed by the publisher.
